# Comment on “Handling persistent cardiac troponin elevation in asymptomatic athletes: The role of macrocomplexes”

**DOI:** 10.1016/j.ahjo.2026.100958

**Published:** 2026-10-14

**Authors:** Sathasivam Sivamalar, Indu Purushothaman, S. Uma, S. Joshua

**Affiliations:** a Scientist, Department of Research, Meenakshi Academy of Higher Education and Research, India; b Department of Research, Meenakshi Academy of Higher Education and Research, India; c Arulmigu Meenakshi College of Nursing, Meenakshi Academy of Higher Education and Research, India; d Meenakshi College of Allied Health Sciences, Meenakshi Medical College Hospital & Research Institute, Meenakshi Academy of Higher Education and Research, India

**Keywords:** Cardiac troponin I, Macrocomplexes, Macrotroponin, Myocarditis, Athletes, PEG precipitation, Troponin elevation, Sports cardiology, Assay interference, Return to play

Dear Editor,

Wernhart et al. [1], measured high-sensitivity cardiac troponin I (cTnI) before and after polyethylene glycol (PEG) precipitation in 13 samples from 12 athletes with persistent cTnI elevation after myocarditis, and propose PEG testing to avoid unnecessary exclusion from sport. The question comes up often in sports cardiology clinics. We think the data support a narrower conclusion.

Most of the recovery values are ceilings rather than measurements. In 11 of 13 samples, cTnI after precipitation was below 5.1 ng/L, and recovery appears to have been calculated by entering 5.1 into the formula [2]. Recovery then depends only on the starting value: any sample above about 29 ng/L with an undetectable post-PEG result falls below 35% by arithmetic alone. These ceilings were then averaged (mean 17.7%, median 17.4%) as if they were measured values. The 0.1% given for sample 13 cannot be right; by the same calculation it is at most 12.9%. The 5.1 ng/L value is the limit of quantification, not of detection, and the assay can usually report lower concentrations. Using them would turn ceilings into estimates. At the 20% cut-off that the authors cite for 92% to 95% specificity, only 7 of the 13 samples could be classified at all [3].

The two samples with measurable post-PEG cTnI deserve more attention. Corrected for the 1:1 dilution, sample 2 retained 37.8 ng/L and sample 6 retained 153.0 ng/L of PEG-soluble cTnI. Both exceed the 26.2 ng/L 99th percentile used in the study, and both athletes are men whose values would also exceed a male-specific limit [4]. Macrocomplexes may well be present in these two, but so, apparently, is free troponin above the reference limit. For a return-to-play decision that residual is the number that matters, and it argues against reassurance in these cases.

The abstract states that macrocomplex formation “seems to be highly prevalent” after myocarditis. Athletes were selected because cTnI stayed elevated while troponin T was normal, and that discordance already points to assay interference. The limitations section rightly says that this selection precludes any estimate of prevalence. The abstract should say the same. Its age summary also does not match the listed ages.

The comparison with troponin T is also weaker than it looks. Troponin T was taken from the medical history and was not measured on the samples tested with PEG, so the two markers may describe different days. A paired measurement would show whether the discordance was still present at the time of testing.

We are also not sure that antitroponin antibodies after myocarditis are purely a laboratory matter. Autoantibodies against cTnI have been reported in myocarditis and dilated cardiomyopathy, and their significance there is unsettled. Calling the result a false positive treats the antibody as clinically inert. Without follow-up of these athletes after return to sport, that remains an assumption.

PEG precipitation is cheap and may help. In its present form, with undetectable post-PEG values, no control samples and no second assay, it seems better suited to starting a stepwise work-up than to ending one.

## CRediT authorship contribution statement

**Sathasivam Sivamalar:** Conceptualization, Data curation, Formal analysis. **Indu Purushothaman:** Visualization, Writing – original draft, Writing – review & editing. **S. Uma:** Visualization, Writing – original draft, Writing – review & editing. **S. Joshua:** Visualization, Writing – original draft, Writing – review & editing.

## Informed consent

Not applicable.

## Ethical approval

Not applicable (Commentary on a previously published article).

## Declaration of Generative AI and AI-assisted technologies in the writing process

Generative AI tools were used only for language refinement and formatting assistance. All scientific interpretation, critical appraisal, and manuscript preparation were performed independently by the authors.

## Funding

None.

## Declaration of competing interest

The authors declare that there are no conflicts of interest.

## Data Availability

No new data were generated or analyzed in this study.
